# Deep Bone Abscess After Pin Tract Infection of an Operatively Treated Supracondylar Humeral Fracture

**DOI:** 10.7759/cureus.101509

**Published:** 2026-01-14

**Authors:** Ahmad G Abdallatif, Hosam Nasr, Andrew Pearse

**Affiliations:** 1 Trauma and Orthopaedics, Worcestershire Acute Hospitals Trust, Worcester, GBR

**Keywords:** abscess clinical presentation, acute trauma care, bone abscess, brodie‘s abscess, early debridement, incision and drainage of abscess, k wire, paediatric orthopedics, supracondylar humeral fracture, upper extremity trauma

## Abstract

Deep bone abscesses are a rare complication following a pin tract infection. This article shows the importance of early detection, surgical management, and microbiology-guided antibiotic therapy to treat the abscess. We aim to highlight the key presenting factors, the radiological signs, and intraoperative features of a deep bone abscess, to facilitate detection in these rare circumstances.

Closed or open reduction and internal fixation with Kirschner wires (K-wires) has become the standard of care for displaced supracondylar fractures. Although pin tract infection (PTI) is the most common complication of K-wire fixation, deep bone abscess following this infection is rare. We are reporting a unique case in which a pin tract infection developed into a deep bone abscess.

An eight-year-old female patient presented with a right supracondylar fracture of the humerus. The fracture was managed with closed reduction and K-wire fixation. This case was complicated by a PTI, which was initially managed with wire removal and oral antibiotics. The infection developed into a deep bone abscess, which required operative incision and drainage, debridement, and intravenous antibiotics.

Septic arthritis, osteomyelitis, and late deep infections are rare complications of pin tract infection, but surgeons must consider them to allow for prompt diagnosis and adequate treatment. Satisfactory long-term outcome of these deep infections can be expected when treated with surgical debridement and intravenous antibiotics.

## Introduction

Pin tract infection (PTI) after closed reduction and internal fixation of supracondylar humeral fractures in the paediatric age range has been reported from 0-8% [1,2,3,4]. Most pin tract infections are superficial and are adequately managed with oral antibiotics and regular dressing. However, the treatment may not be straightforward and in some cases may develop deep abscesses [5].

There is no agreement in the current literature about grading the severity of pin tract infections [5]. Clint et al classify PTIs into good, bad, and ugly based on clinical signs, including pain, irritation, and erythema [6]. Many authors propose the Checketts-Otterburn PTI classification as a guide for treatment planning. According to this classification, the infections are divided into two categories, minor (grades 1-3) and major (grades 4-6) [7].

A few studies have reported the management of deep PTI, and most authors have different approaches in treating this rare complication [8]. Long-term results and functional outcomes after deep infection treatment are also unclear and have not been investigated before in the literature [9].

In our study, we report a rare case with a deep bone abscess following a PTI of a supracondylar fracture of the humerus after treatment. We reported the management of this complication and outcome after six months of follow-up.

## Case presentation

**Figure 1:**
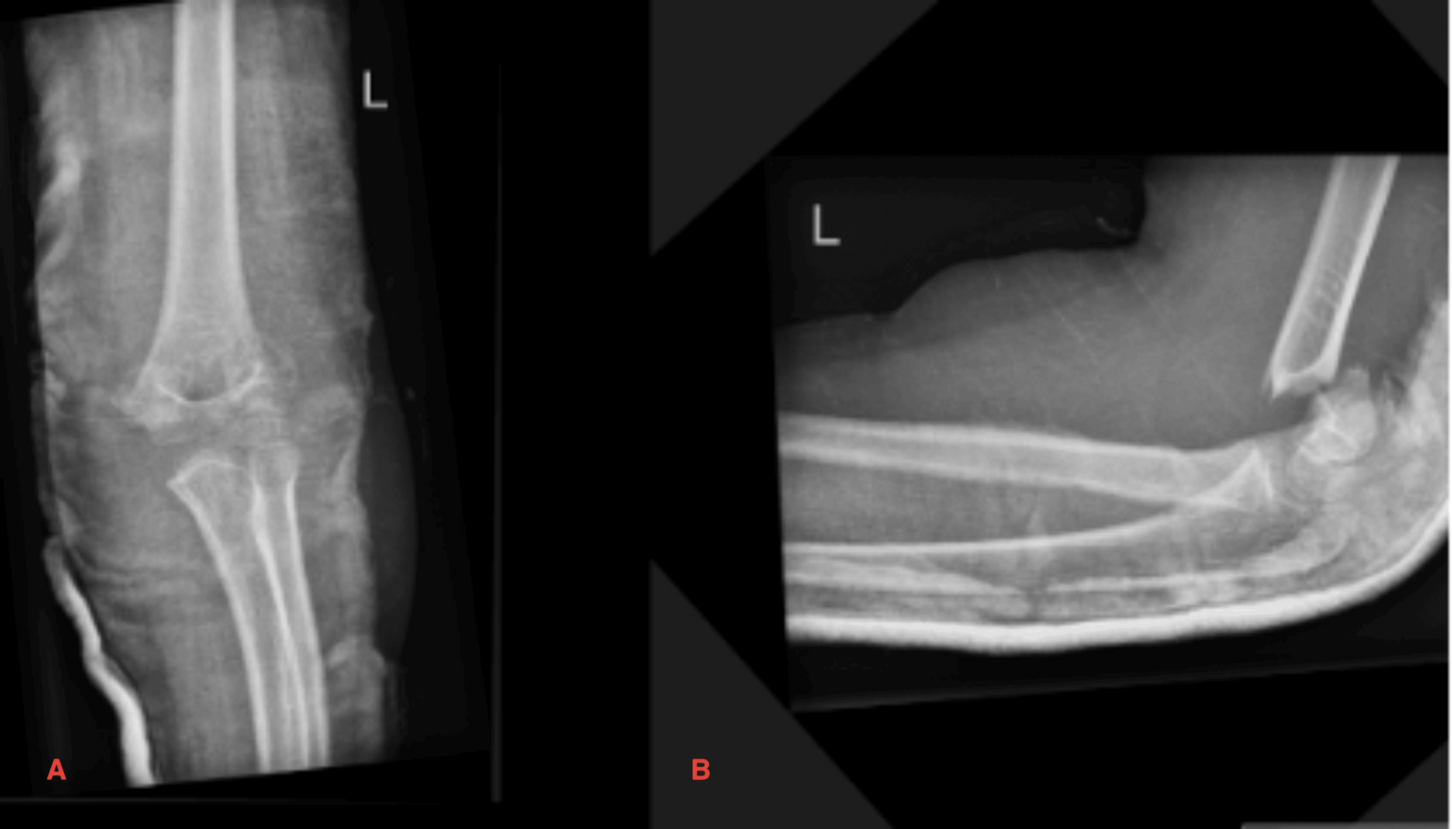
Left displaced supracondylar humeral fracture A - AP view; B - Lateral view

**Figure 2:**
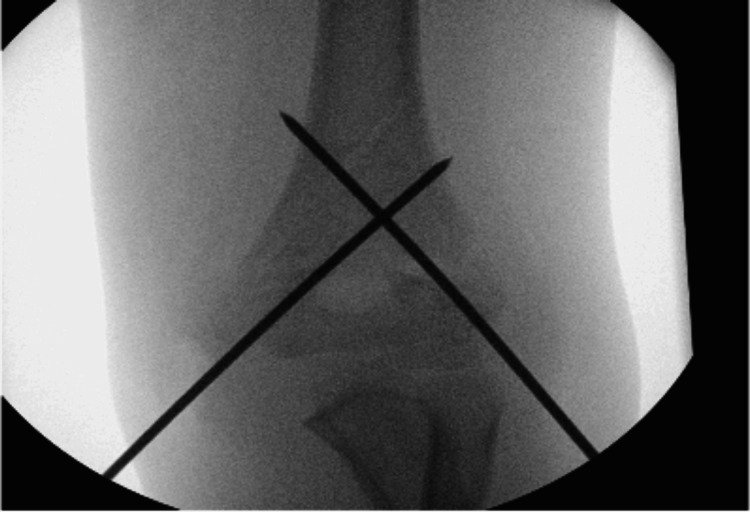
Intraoperative image of closed reduction with percutaneous pinning with 2 mm K-wires

An eight-year-old female patient presented to the Accident & Emergency (A&E) department with a closed supracondylar humerus fracture (Figure 1). The patient was admitted on the same day and had an operation the next day in the form of a closed reduction and internal fixation with two crossed 2.0 mm K-wires (Figure 2). The operative time was 45 minutes, and no intraoperative complications were encountered. The patient was discharged the following day with an outpatient follow-up appointment booked for a week later.

**Figure 3:**
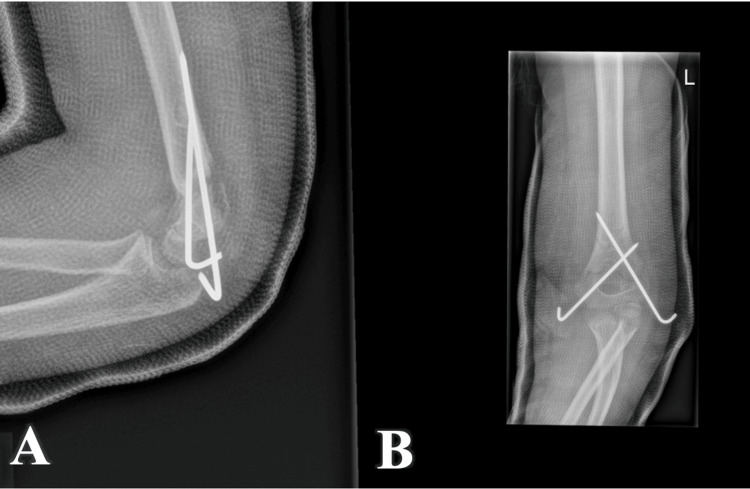
Follow-up radiograph one week after the procedure. A - Lateral view of the elbow, with K-wires in situ; B - Anteroposterior view of the elbow, with K-wires in situ.

After one week, the patient had the first postoperative visit. During this visit, the patient had a follow-up X-ray (Figure 3), which was satisfactory. The patient also had a new dressing applied, with no concern about wound infection.

The second postoperative visit was three weeks later, when the patient had the wires removed in the clinic, and the patient was discharged without concern about wound infection.

After six weeks, the patient had another appointment, as the patient’s parents were concerned about an infection in the patient’s elbow. On examination, the patient had signs of a PTI, and oral co-amoxiclav was prescribed for three weeks. A follow-up radiograph was requested. The radiograph showed complete healing at the fracture site with good alignment. The radiograph demonstrated a sign of excessive periosteal reaction and a bone cyst, prompting the need for further evaluation in the form of an MRI scan.

**Figure 4:**
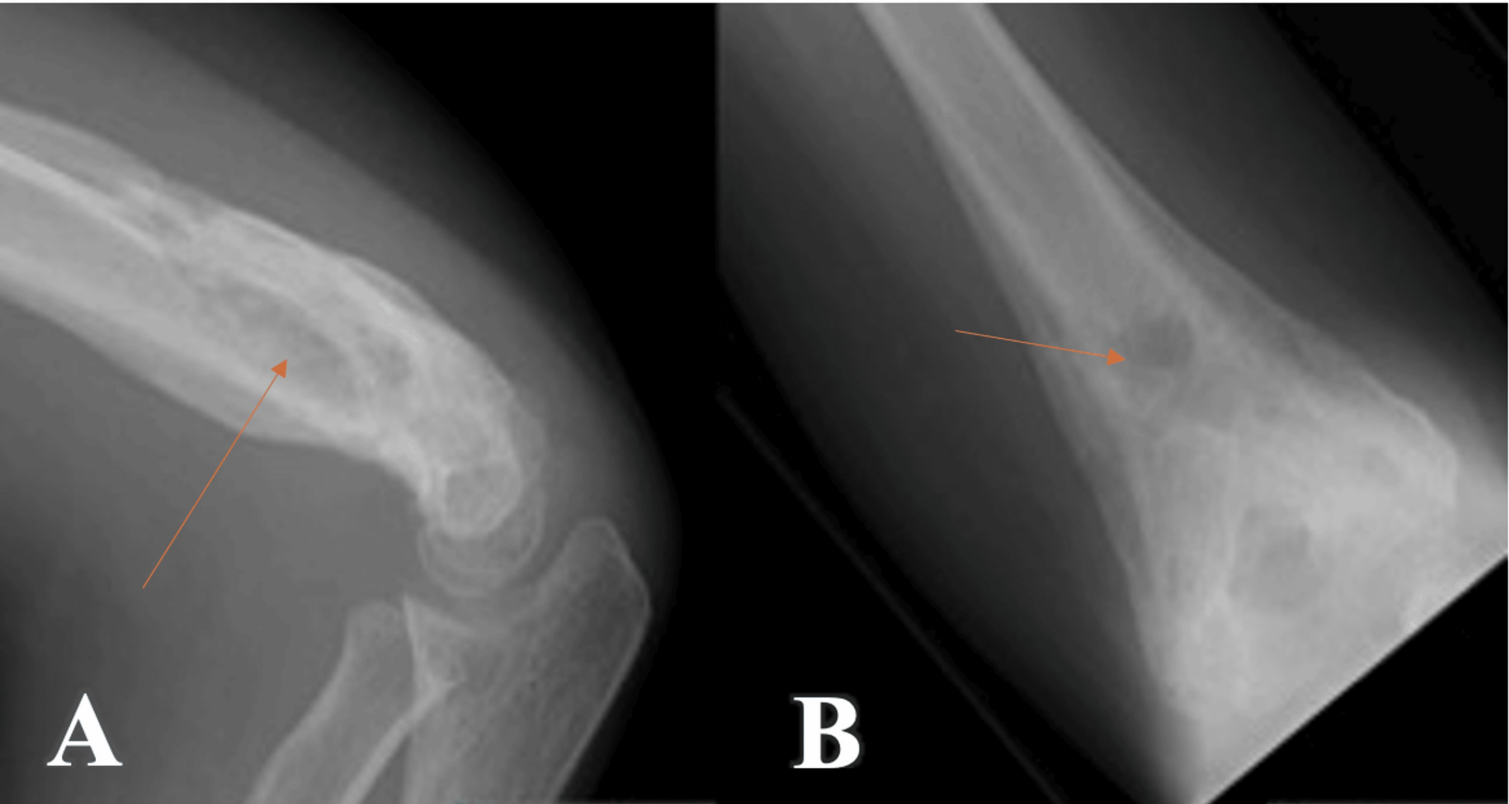
10 weeks postoperative radiograph, with radiological signs of deep infection (excessive periosteal reaction, with central bone cyst) A - Lateral view of the elbow; B - Anteroposterior view of the elbow

**Figure 5:**
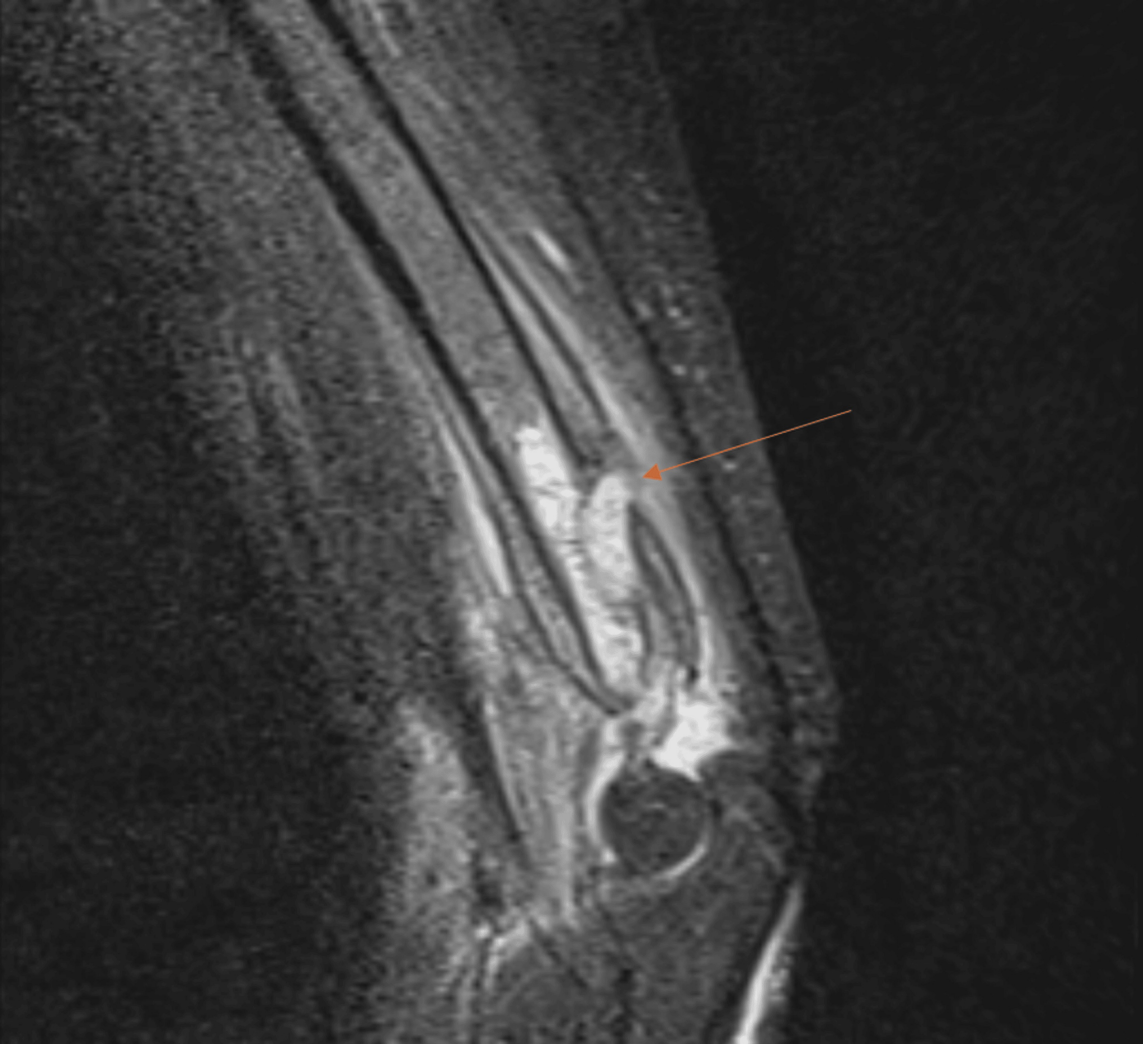
MRI shows a deep bone abscess and posterior sinus Deep bone abscess is shown with the arrow.

At week 10, the signs of PTI did not improve, and the patient started having stiffness at the elbow with a gradual loss of range of movement, chronic pain, and hotness. Based on the above findings, a follow-up radiograph and an MRI scan were requested. The radiograph shows excessive periosteal reactions and a bone cyst (Figure 4), and an MRI (Figure 5) confirmed the presence of a deep abscess with a posterior bone sinus. Blood tests showed elevation of C-reactive protein (CRP) (24 mg/dL, normal <4 mg/dL).

**Figure 6:**
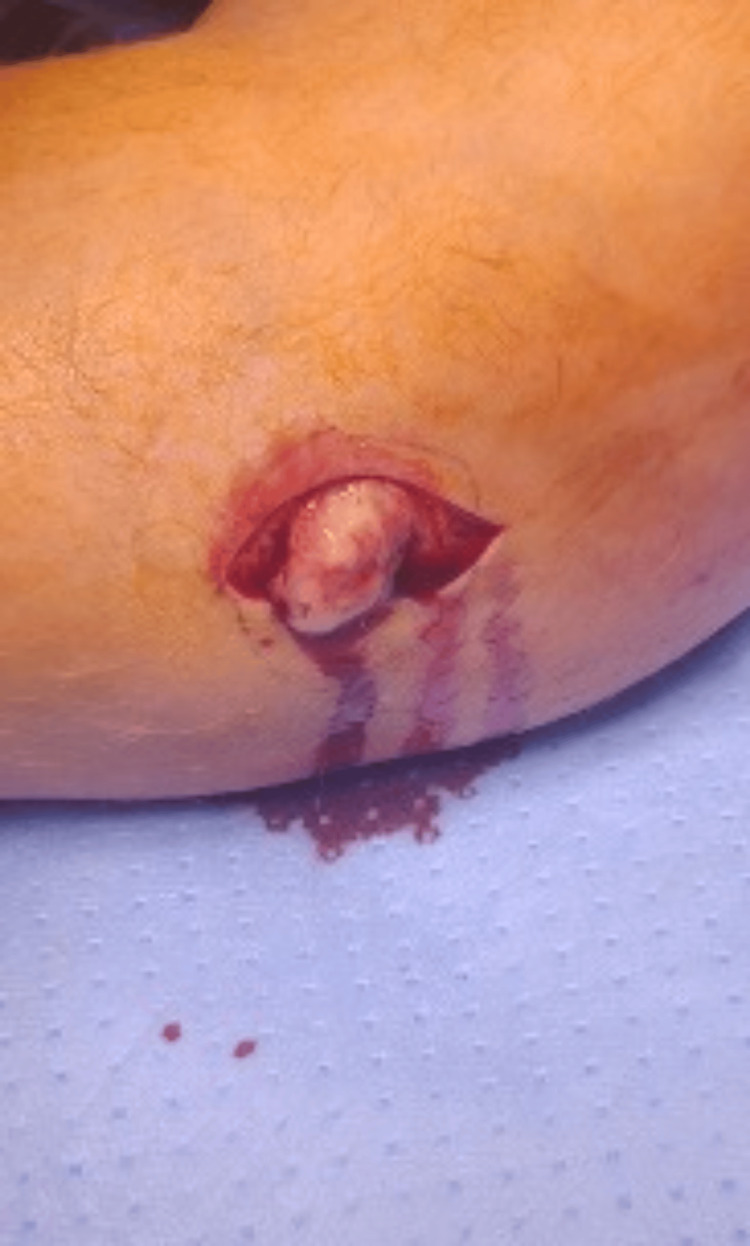
Intraoperative image showing drainage of the deep bone abscess

A decision was made for a surgical intervention in the form of deep debridement, opening of the sinus tract, and debridement of the cavities with decompression of the abscess, debridement, and washout (Figure 6).

During the surgical procedure, samples were taken from the medullary canal as well as from the surrounding tissues. The microbiology samples were sent for culture and sensitivity tests, with a request for the microbiology team to detect the proper postoperative antibiotic regimen.

Enterobacter was isolated from the culture, and based on the microbiology team’s request, the patient commenced on co-amoxiclav 480 mg orally for 6 weeks. The patient was discharged with a weekly follow-up appointment at an outpatient clinic.

**Figure 7:**
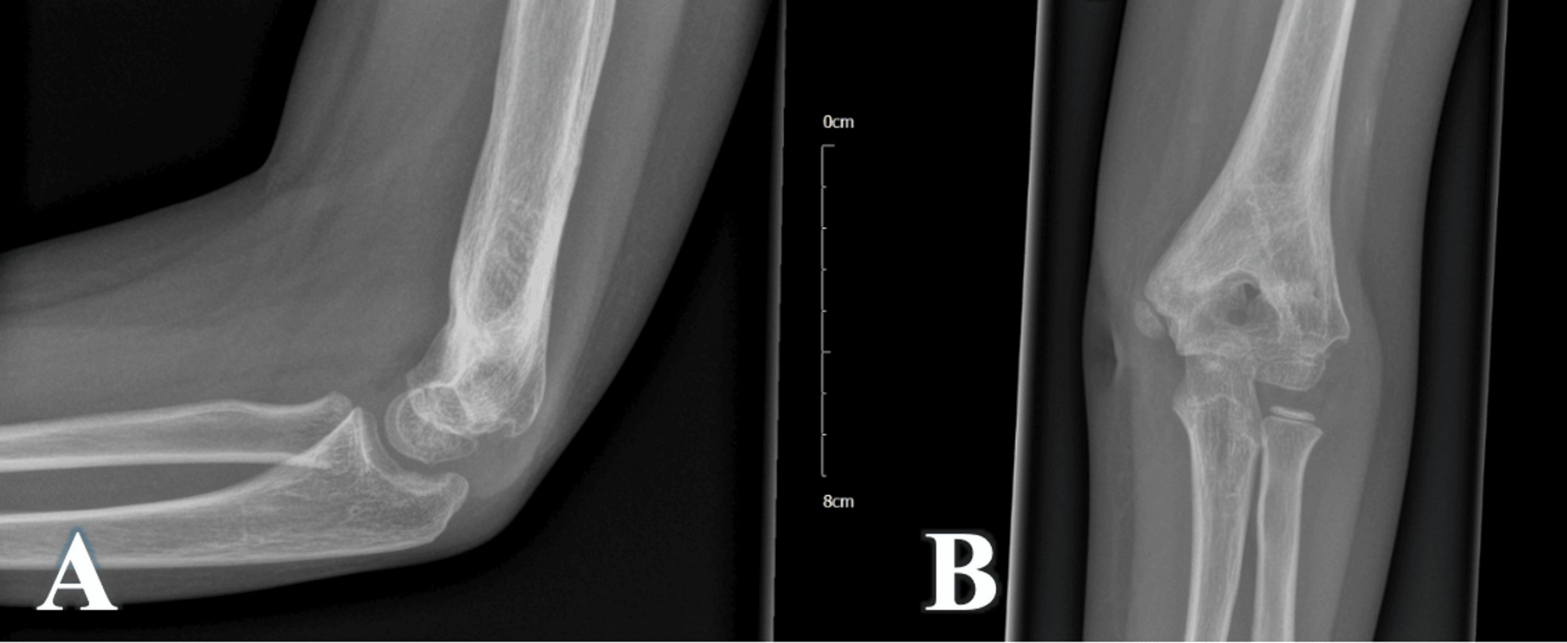
Final follow-up radiographs of the elbow, showing complete resolution A - Lateral view of the elbow; B - Anteroposterior view of the elbow

Over the next eight weeks, the patient's CRP continued to reduce from 24 to normal at the last two follow-up visits. The patient’s follow-up radiographs also showed complete obliteration of all previous sinuses (Figure 7). Clinically, the swelling, redness, and signs of PTI completely resolved. Under the care of the physiotherapy team, the patient returned to a full range of movements and back to her sporting activities by the last follow-up visit.

## Discussion

Deep bone infection or osteomyelitis is a rare complication after a pin tract infection. Different ways of managing this complication were described, but surgical intervention was always recommended [10]. The most important thing is the high index of suspicion of this unique complication, especially with the persistence of pin tract infection after 7 days of oral antibiotics [5]. There is no place for conservative treatment with the presence of bone infection; most of the time, it is surrounded by a thick periosteum, which decreases the effect of systemic antibiotic treatment. Early surgical intervention is the key to successful treatment and achieving the best outcome [8].

Parameswaran et al. [11] recommended debridement of the osteomyelitis cavities and leaving antibiotic beads in the tracts, while Moroni et al. [12] filled these cavities with calcium phosphate pellets impregnated with antibiotics.

Although filling abscess cavities with antibiotic beads or calcium phosphate impregnated with antibiotics is very effective, both have their own complications [11,12]. In our case, we achieved a full curative status with only deep debridement and decompression of the cavities without the need to fill these cavities with any foreign materials.

Active involvement of the microbiology team is crucial for effective treatment and achieving good results. All patients with deep bone abscesses still need to have an antibiotic cover after surgical debridement. The type of antibiotic, as well as the duration, is different based on isolated organisms as well as personal factors [13]. Although many studies recommended parenteral antibiotic coverage after surgical intervention [3,7,8], in our case, complete recovery was achieved with oral antibiotics.

More studies are still needed to standardise the PTI treatment and time frame to initiate a surgical intervention, potentially focusing on the length of antibiotic treatment required as well as thresholds on imaging to consider operative intervention.

## Conclusions

Most infections after pinning of supracondylar humerus fractures are superficial and can be managed with pin removal, oral antibiotics, and local wound care. Septic arthritis, osteomyelitis, and late deep infections are rare complications of closed reduction with percutaneous pinning, but surgeons must consider them to allow for prompt diagnosis and adequate treatment. Having a high index of suspicion for the development of a bone abscess in the presence of a sinus, ongoing pain, and no response to antibiotics will facilitate early diagnosis of this complication. Satisfactory long-term outcomes of these deep infections can be expected when treated aggressively with surgical debridement and intravenous antibiotics.

## References

[1] Skaggs DL, Sankar WN, Albrektson J, Vaishnav S, Choi PD, Kay RM (2008). How safe is the operative treatment of Gartland type 2 supracondylar humerus fractures in children?. J Pediatr Orthop.

[2] Bashyal RK, Chu JY, Schoenecker PL, Dobbs MB, Luhmann SJ, Gordon JE (2009). Complications after pinning of supracondylar distal humerus fractures. J Pediatr Orthop.

[3] Iobst CA, Spurdle C, King WF, Lopez M (2007). Percutaneous pinning of pediatric supracondylar humerus fractures with the semisterile technique: the Miami experience. J Pediatr Orthop.

[4] Devkota P, Khan JA, Acharya BM (2008). Outcome of supracondylar fractures of the humerus in children treated by closed reduction and percutaneous pinning. JNMA J Nepal Med Assoc.

[5] Syngouna SA, Mitsikostas PK, Fandridis E, Triantafyllopoulos IK Pin site infections in pediatric population, microbiology, treatment and long-term functional disability. J Res Prac Musculoskel Sys.

[6] Clint SA, Eastwood DM, Chasseaud M, Calder PR, Marsh DR (2010). The "Good, Bad and Ugly" pin site grading system: a reliable and memorable method for documenting and monitoring ring fixator pin sites. Injury.

[7] Parikh SN, Lykissas MG, Roshdy M, Mineo RC, Wall EJ (2015). Pin tract infection of operatively treated supracondylar fractures in children: long-term functional outcomes and anatomical study. J Child Orthop.

[8] Perone A, Graham HK, Krajbich JI (1988). Management of displaced extension-type Supracondylar fracture of the humerus in children. J Bone Joint Surg Am.

[9] Boy D, Aronson DD (1992). Supracondylar fracture of the humerus: a prospective study of percutaneous pinning. J Pediatr Orthop.

[10] Checketts RGMA, Otterburn M. DeBastiani AGAA, Goldberg DE (2000). Pin track infection and the principles of pin site care. Orthofix External Fixation in Trauma and Orthopedics.

[11] Parameswaran AD, Roberts CS, Seligson D, Voor M (2003). Pin tract infection with contemporary external fixation: how much of a problem?. J Orthop Trauma.

[12] Moroni A, Toksvig-Larsen S, Maltarello MC, Orienti L, Stea S, Giannini S (1998). A comparison of hydroxyapatite-coated, titanium-coated, and uncoated tapered external-fixation pins. An in vivo study in sheep. J Bone Joint Surg Am.

[13] Kocher MS, Kasser JR, Waters PM (2007). Lateral entry compared with medial and lateral entry pin fixation for completely displaced supracondylar humeral fractures in children. A randomized clinical trial. J Bone Joint Surg Am.

